# Measuring and Exploring Children’s Health Literacy in The Netherlands: Translation and Adaptation of the HLS-Child-Q15

**DOI:** 10.3390/ijerph18105244

**Published:** 2021-05-14

**Authors:** Marla T. H. Hahnraths, Monique Heijmans, Torsten M. Bollweg, Orkan Okan, Maartje Willeboordse, Jany Rademakers

**Affiliations:** 1 Department of Family Medicine, Care and Public Health Research Institute (CAPHRI), Maastricht University, P.O. Box 616, 6200 MD Maastricht, The Netherlands; maartje.willeboordse@maastrichtuniversity.nl; 2 Netherlands Institute for Health Services Research (NIVEL), P.O. Box 1568, 3500 BN Utrecht, The Netherlands; m.heijmans@nivel.nl (M.H.); j.rademakers@nivel.nl (J.R.); 3 Centre for Prevention and Intervention in Childhood and Adolescence, Faculty of Educational Science, Bielefeld University, Universitätsstrasse 25, 33615 Bielefeld, Germany; torsten.bollweg@uni-bielefeld.de (T.M.B.); orkan.okan@uni-bielefeld.de (O.O.); 4 Interdisciplinary Centre for Health Literacy Research, Bielefeld University, Universitätsstrasse 25, 33615 Bielefeld, Germany

**Keywords:** health literacy, child, surveys and questionnaires, Netherlands, assessment

## Abstract

As health literacy (HL) is hypothesized to develop throughout life, enhancement during childhood will improve HL and health during life. There are few valid, age-appropriate tools to assess children’s HL. The German-language European Health Literacy Survey Questionnaire Adapted for Children (HLS-Child-Q15-DE) is a self-report questionnaire adapted from the adult European Health Literacy Survey Questionnaire. This study aims to translate the HLS-Child-Q15 to Dutch and explore the sample’s HL distribution. The HLS-Child-Q15-DE was translated following WHO guidelines and administered digitally to 209 Dutch schoolchildren (eight-to-eleven-year-olds). Its psychometric properties were assessed and the sample’s HL distribution was explored by demographic characteristics. The HLS-Child-Q15-NL had high internal consistency (α = 0.860) and moderate to strong item-total correlations (mean = 0.499). For 6 of the 15 items, >10% of participants answered “do not know”, indicating comprehension problems. Higher HL scores were observed for ten-to-eleven-year-olds (compared with eight-to-nine-year-olds; *p* = 0.021) and fourth-grade students (compared with third-grade; *p* = 0.019). This supports the idea that HL evolves throughout life and the importance of schools in this process. With the HLS-Child-Q15-NL, a Dutch measurement instrument of children’s HL is available, although it needs further tailoring to the target group. More research is needed to decrease comprehension problems and to investigate retest reliability and construct validity.

## 1. Introduction

Health literacy (HL) is defined as “people’s motivation, knowledge and competences to access, understand, appraise, and apply health information to make judgements and take decisions in everyday life concerning healthcare, disease prevention and health promotion to maintain or improve quality of life throughout life” [1]. Multiple studies have demonstrated positive associations between HL and health outcomes in adults (e.g., diabetes outcomes, hospitalizations) [2,3]. Over the last years, children’s HL has received increasing attention. As HL is hypothesized to be a skill that develops throughout life, enhancing it at a young age (when various prerequisite competencies for HL also evolve) will likely result in improved HL and health outcomes later in life [4,5,6]. Despite the growing interest, little knowledge is available on children’s HL; partly due to the fact that until recently, children and adolescents generally have been overlooked in health research [7]. The scarcity of valid, age-appropriate instruments to assess children’s HL further contributes to this research gap [8,9,10].

The European Health Literacy Survey Questionnaire (HLS-EU-Q47) is a 47-item measurement instrument to assess HL in adults (15+ years). It was developed and validated by the HLS-EU Consortium to compare HL across eight European countries [1,11,12]. An age-adapted version of the HLS-EU-Q47 was developed and tested for German-speaking children aged 9–10 years. The development and validation process, resulting in the 15-item HLS-Child-Q15-DE, is presented elsewhere [13,14]. In a first study investigating HLS-Child-Q15-DE’s psychometric properties, good internal consistency was demonstrated [15]. Since its development, efforts are being made to translate the HLS-Child-Q15 into other languages (e.g., English, French, Portuguese [16]). Currently, no Dutch translation of the HLS-Child-Q15 (or any other Dutch-language instrument to assess children’s HL) is available. To be able to assess Dutch children’s HL and to make comparisons with other countries, the present study was initiated. More specifically, the study aims to:

1. Translate the HLS-Child-Q15 into Dutch;
2. Test the Dutch HLS-Child-Q15 in a sample of Dutch primary school children:
  - To verify its internal consistency and investigate data quality;
  - To explore the distribution of children’s HL over various demographic characteristics (sex, age, grade, ethnicity, socioeconomic status (SES)).

## 2. Materials and Methods

### 2.1. Translation and Adaptation Process

For translating and adapting the HLS-Child-Q15, a systematic five-step approach conforming to WHO guidelines was followed, including forward translation, expert panel meeting, backward translation, pre-testing/cognitive interviewing, and consensus about the final version [17]. Two independent professional Dutch translators performed the forward translation. The expert panel included both translators and four professionals/researchers with expertise in HL, child development, health promotion, and development of measurement instruments. During the expert panel meeting, discrepancies between the two translations were discussed and resolved. This resulted in agreement upon a single translation of the HLS-Child-Q15, which was then translated back to German by a third independent professional translator. As there were only minor textual discrepancies between the backward translation and the original version, it was concluded that the Dutch translation was satisfactory and ready for pre-testing. Pre-testing was done in individual cognitive interviews in a sample of ten children aged 9–10 years (five male, five female). All 15 translated HLS-Child-Q15 items were discussed and participants were asked to think aloud, contemplating about their interpretations and the items’ meanings and phrasing. Furthermore, the response categories were discussed, and the interviewer asked questions about the questionnaire’s general comprehensiveness. The cognitive interviews did not lead to major alterations in the translated HLS-Child-Q15, although rephrasing of some items was needed (e.g., “to find out” instead of “to learn”, and “on which moment” instead of “when”). Most children comprehended the items as intended, which demonstrated adequate face validity of the questionnaire. The final version of the instrument (HLS-Child-Q15-NL) is attached as Appendix A.

### 2.2. Questionnaire Administration

After translation, the final version of the HLS-Child-Q15-NL was incorporated in a larger online questionnaire on children’s health, well-being, and dietary and physical activity behaviors. Children filled out the questionnaire in class during school hours; they were instructed to answer the questions individually and to ask questions to available researchers if something was unclear. Filling out the complete questionnaire (39 multi-item questions) took about 30 min. The digital format did not allow participants to skip questions, but for every item, it was possible to select the “do not know” option.

### 2.3. Participants

The present study is part of a larger research project involving twelve primary schools in Limburg, a province in the south of the Netherlands. This project investigates the effects of school-based health-promoting initiatives on children’s health and well-being (e.g., body mass index, dietary and physical activity behaviors). Data collection for the current study was incorporated in the projects’ baseline measurements.

All students of grades three and four (aged 8-11 years; corresponding to study years five and six in the Netherlands) of these twelve schools (n = 436) were eligible to participate in the present study; there were no further inclusion or exclusion criteria. Recruitment for the study was done via brochures for parents, which contained information about the research aims, procedures and data handling. Furthermore, researchers visited classrooms to inform children about the project and encourage them to participate. After school time, parents could ask questions to the researchers. All participating children were required to hand in a completed informed consent form, signed by both parents/guardians. The need for ethical approval for the overall research project was waived by the Medical Ethics Committee Zuyderland in Heerlen (METC-Z no. METCZ20190144). The project was registered in the ClinicalTrials.gov database on 9 December 2019 (NCT04193410).

### 2.4. Measures

#### 2.4.1. Covariates

Children’s age and sex were collected via the educational board’s database. A digital parental questionnaire was used to obtain information about the children’s SES and ethnicity. SES was calculated as the mean of standardized scores on maternal and paternal educational level [18]. The mean scores were categorized into low, middle, and high SES scores based on tertiles. Children’s ethnicity was determined by parental country of birth and divided into (1) Dutch, (2) Western (i.e., all other European countries (excluding Turkey), and North America, Japan, Indonesia, and Oceania), and (3) non-Western [19]. If at least one parent was born in a Western (other than the Netherlands) or non-Western country, the child’s ethnicity was labelled Western or non-Western, respectively.

#### 2.4.2. Outcomes

HL was measured using the HLS-Child-Q15-NL, which contained 15 items assessing the child’s perceived ease or difficulty in finding, understanding, appraising, and applying health information. All items were phrased “How easy or difficult is it for you to…”. Responses were given on a four-point Likert scale (i.e., “very difficult”, “difficult”, “easy”, “very easy”). Additionally, a “do not know” response category was incorporated. Higher scores indicate perceived ease in dealing with health information (i.e., higher HL).

### 2.5. Statistical Analyses

To maximize comparability with the original development study, similar statistical protocols were used [15]. All analyses were performed using IBM SPSS Statistics for Windows (version 25.0). Due to the digital questionnaire’s nature, participants could not skip questions, resulting in no true missings in the collected data. However, participants could select the “do not know” option, and questionnaires with ≥14 times “do not know” (maximum missing rate of 80%) were excluded from analyses, as these participants either had no intention of filling in the questionnaire or were unable to do so due to language problems. Since the literature does not provide hard cutoff points for missing values, the cutoff point of 80% is arbitrary and based on agreement between the Dutch researchers and the developers of the original German questionnaire. For other participants, “do not know” responses were handled as missing data.

To investigate the instrument’s data quality, each item was examined separately by looking at the mean (with standard deviation (SD)), percentage of “do not know” answers, proportion of maximum agreement (i.e., item difficulty), and variance. If >10% of participants answered “do not know” for an item, this was interpreted as indicative of comprehension problems. For proportion of maximum agreement, the percentage of participants selecting the maximum possible response option (i.e., “very easy”) was assessed, with desirable values between 20% and 80% [20]. As a second measure of differentiation, item variance was assessed (higher values are desirable) [21]. Internal consistency (i.e., degree of similarity between items) was measured as Cronbach’s alpha coefficient and Spearman Brown split-half reliability coefficient. Values of ≥0.70 indicate sufficient internal consistency [22]. Inter-item correlations and corrected item-total correlations (ITCs; correlation between an item and the overall score formed by all other items) were calculated. A correlation r ≥ 0.50 is considered strong, r ≥ 0.30 moderate, and r ≥ 0.10 weak [23].

Furthermore, the sample’s overall HL scores were calculated. No HL scores were calculated for respondents with >3 missing responses (maximum missing rate of 20%), meaning that HL scores were calculated for a more restricted sample than the sample used to assess the instrument’s data quality (where a maximum missing rate of 80% was used) [12,15]. For maximum transparency, three HL estimates were provided: (1) overall mean scores (calculated by dividing the sum of valid responses by the total number of valid responses); (2) quintiles (first quintile = “lowest HL”, second to fourth quintile = “medium HL”, fifth quintile = “highest HL”); and (3) HL levels corresponding to the HLS-EU-Q47 health literacy indices [12]. For the latter, mean overall HL scores were transformed from a range of one to four to a unified metric with a minimum of zero (least possible HL score) and a maximum of 50 (best possible HL score). Subsequently, HL estimates were categorized using four previously defined levels [12]; “inadequate” (0–25), “problematic” (>25–33), “sufficient” (>33–42), and “excellent” (>42–50).

Normality of the distribution of overall mean HL scores in the sample was checked using histograms. Independent-sample t-tests and one-way ANOVA were used to explore the HL distribution in the sample by sex, age, grade, ethnicity, and SES, while Welch tests were used in case the Levene’s test showed that variances were significantly different. For all analyses, a two-sided *p*-value ≤0.05 was considered statistically significant.

## 3. Results

### 3.1. Sample

Of the 436 students eligible for study participation, parental consent was obtained for 215 students (49.3%). Six participants were excluded from analyses due to having selected “do not know” ≥14 times, resulting in 209 participants included in the present study. Slightly less than half were male (46.4%) and the sample’s mean age was 9.71 years (SD: 0.68). The majority of the sample had a Dutch background (95.1%) and a SES in the highest tertile (49.1%). Table 1 reports the sample characteristics.

### 3.2. Psychometric Properties

Table 2 presents an overview of the 15 items tested and the statistics from item analyses.

*Missing values.* The percentage of participants selecting the “do not know” response ranged from 5.3% (item 15) to 22.5% (item 1), with a mean of 9.8% (SD: 4.77) per item. Six items had a missing rate >10%.

*Item difficulty.* Item difficulty parameters ranged from 14.8% (item 1) to 58.6% (item 15). One item (item 1) was observed in the “difficult” answer spectrum (item difficulty parameter <20%). All other items had “medium” difficulty (item difficulty parameter between 20% and 80%).

*Variance.* Standard deviations ranged from 0.627 (item 15) to 1.08 (item 8), with an average SD of 0.847 for all items.

*Internal consistency.* Cronbach’s alpha coefficient (α = 0.860; 95% CI (0.815; 0.898)) and Spearman Brown split-half reliability coefficient (r = 0.838; 95% CI (0.497; 0.947)) indicated high internal consistency. Inter-item correlations ranged from 0.009 (between item 4 and 9) to 0.558 (between item 4 and 11). No items had inter-item correlations <0.30 with all other items (Table S1). ITCs ranged from 0.412 to 0.654, with an average ITC of 0.499. No items had an ITC <0.30, nine items had an ITC between 0.40 and 0.50, and six items an ITC >0.50.

### 3.3. Distribution of HL Levels

HL scores were calculated for participants with ≤3 missing responses, resulting in mean scores for 180 of 209 participants (86.1%).

*Overall mean HL scores.* Overall mean scores ranged from 1.53 to 4.00, indicating that respondents used nearly the complete response range (1 = “very difficult” to 4 = “very easy”). The sample’s mean score was 3.14 (SD: 0.465). Table 3 shows the HL distribution based on quintiles, with the categories “lowest HL” (mean score ≤ 2.73), “medium HL” (mean score > 2.73 and < 3.53), and “highest HL” (mean score ≥ 3.53).

*HL scores based on HLS-EU-Q47 indices.* The sample’s mean HL score based on the HLS-EU-Q47 indices was 35.68 (SD: 7.76). Scores ranged from 8.89 to 50.00. When looking at the HL distribution across the four levels (Table 3), most participants had a “sufficient” HL level (45.6%), while an “inadequate” HL level was least frequently observed (9.4%).

Independent-sample t-tests indicated that ten-to-eleven-year-olds had significantly higher HL scores (3.20 ± 0.463) compared to eight-to-nine-year-olds (3.04 ± 0.453), *t*(178) = −2.33, *p* = 0.021. Additionally, HL scores for students from grade four were significantly higher (3.21 ± 0.455) compared with students from grade three (3.05 ± 0.465), *t*(178) = −2.36, *p* = 0.019. No significant differences in overall mean HL scores were found for other background characteristics (Table 4).

## 4. Discussion

In the present study, the HLS-Child-Q15 was translated to Dutch and subsequently tested in a sample of primary school children in Limburg, the Netherlands. Furthermore, the sample’s HL distribution was explored across various demographic variables.

### 4.1. Translation, Adaptation, and Psychometric Properties

Psychometric analyses revealed high internal consistency and moderate to strong ITCs, with slightly higher values than observed in the German sample [15]. During questionnaire administration, various participants asked questions, indicating problems with item interpretation. Participants tended to answer based on their knowledge and experience (e.g., “I know what to do to relax” or “I relax often”), instead of based on their perceived ease or difficulty to deal with health information. Similar problems were reported in the qualitative pre-test of the HLS-Child-Q15-DE [13]. This might indicate that HL is a difficult concept for children to grasp, and that the HLS-Child-Q15 needs further tailoring to the target group (e.g., by simplifying item phrasing or adding pictures/example items). Additionally, adult guidance might be beneficial for successful administration, although excessive adult interference should be avoided to minimize influencing children’s answers. A general supervision protocol might be helpful to ensure adequate adult guidance. 

The relatively high percentage of “do not know” answers could also be due to interpretation problems. Possibly, children interpreted “do not know” as “I do not know how to do that” (e.g., “I do not know what to do to relax”) or “I never do that” (e.g., “I never find out what to do to relax”). Although further research is needed to gain more specific insight into any problems, the “do not know” category could be further specified to “I do not understand the question” to avoid misinterpretation. Administration procedures could also have influenced children’s responses. In the present study, the HLS-Child-Q15 was included at the end of a questionnaire assessing diverse aspects of health and well-being (total administration time approximately 30 min). Possibly, this questionnaire was too long for children and therefore decreased their ability to adequately fill in the HLS-Child-Q15. Administration of the HLS-Child-Q15-NL in isolation would therefore be beneficial to investigate if interpretation problems persist.

To further improve the HLS-Child-Q15-NL, it might be useful to specifically look at the first item (“How easy or difficult is it for you to find out how to recover quickly when you have a cold?”), which had the highest percentage of “do not know” answers and was the only item within the “difficult” answer spectrum. This could be due to formulation and interpretation problems, but it could also be that the item does not connect adequately to children’s everyday lives, as parents might be responsible for this task instead of children themselves. This further supports the notion that the current HLS-Child-Q15-NL is not yet optimally tailored to the target group.

### 4.2. Distribution of HL Levels

The Dutch mean HL score (3.14) is slightly lower than the German score (3.34) [15], which could be due to actual HL differences, although other factors (e.g., differences in setting, administration and item interpretation) might also have played a role. The present sample’s significantly higher HL scores for older participants and for participants from grade four as compared with grade three support the idea that HL is a dynamic concept developing throughout life. Education might have important influences on children’s HL development; making schools powerful settings in this process. With regard to HL scores based on the HLS-EU-Q47 indices, the present sample’s mean score (35.68) is lower than the score previously observed in Dutch adults (37.06), which might be another indicator of evolving HL throughout life [12]. However, as both scores were acquired using different instruments (HLS-Child-Q15-NL and HLS-EU-Q47, respectively) it is not known if and how they can be compared, and more research is necessary before any conclusions can be drawn.

### 4.3. Study Limitations and Implications for Further Research

The present study has several limitations. The fact that all participants are from the same area in the Netherlands (i.e., Limburg) and that information about the non-response group is lacking limits the results’ generalizability. Concerning the psychometric analyses, the sample size was fair, with a total of 180 participants for whom HL scores were calculated (subject-to-item ratio = 12) [24,25]. With regard to the analyses of the HL distribution across demographic characteristics, however, the relatively low number of participants might have limited the ability to detect significant differences. Additionally, due to the present study’s practical constraints, the questionnaire was only administered once, making it impossible to assess test retest reliability. Furthermore, as no other HL-related questions were included in the questionnaire, it was impossible to investigate the instrument’s discriminant and convergent validity. Lastly, children’s lack of experience in relation to the addressed tasks might decrease their answers’ validity, although this needs further investigating.

Further research within a larger, more diverse sample (e.g., in terms of ethnicity, educational quality, and/or SES), using repeated assessments and other HL-related questions is necessary to investigate the results’ generalizability and the instrument’s test retest reliability and discriminant and convergent validity. Multilevel analyses could furthermore clarify the effects different school environments might have on children’s HL. Further investigating children’s interpretation of the “do not know” category would provide more insight into any interpretation problems. Lastly, experimenting with simplified item formulation and various layouts (e.g., adding pictures/examples) and administration methods (e.g., adult guidance, providing solely the HLS-Child-Q15) is needed to further tailor the HLS-Child-Q15 to the target group.

## 5. Conclusions

The HLS-Child-Q15-NL is a promising instrument to measure children’s HL. The questionnaire has high internal consistency, and ITCs are moderate to strong. However, the relatively high percentage of “do not know” responses and the number of questions asked during questionnaire administration indicated comprehension problems. Further refinement of the instrument is necessary to increase its suitability for the target group. Additionally, adult guidance might be beneficial for successful administration, although this should be done with care to avoid influencing children’s answers.

In the present sample, HL scores were higher for older participants and participants from grade four as compared with grade three, which supports the idea that HL evolves throughout life. Education (and therefore schools) can play an important role in HL development, although more research is needed to further investigate this potential working mechanism. The present study’s efforts are first steps towards HL measurement in Dutch children and they increase comparability with other countries.

## Figures and Tables

**Table 1:** Sample characteristics (n = 209).

Characteristic		
	n	%/mean (± SD)
Sex (% boys)	209	46.4
Age (years)	209	9.7 (0.682)
8–9 years	78 ^1^	37.3
10–11 years	131 ^2^	62.7
Grade	209	
Grade three		45.0
Grade four		55.0
Ethnicity	162	
Dutch		95.1
Western		2.5
Non-Western		2.5
SES (%) ^3^	163	
Lowest tertile		20.2
Middle tertile		30.7
Highest tertile		49.1

Abbreviations: SD, standard deviation; SES, socioeconomic status. ^1^ Eight-year-olds (n = 4) and nine-year-olds (n = 74). ^2^ Ten-year-olds (n = 110) and eleven-year-olds (n = 21). ^3^ Due to clustering of SES scores around several scores, the tertile group sizes are unequal.

**Table 2:** Data quality and corrected item-total correlations of the HLS-Child-Q15-NL (n = 209).

Question	“How Easy or Difficult Is It for You to…”	Mean	SD	“Do Not Know” (%)	Proportion of Maximum Agreement (%) ^1^	Variance	ITC
1	find out how to recover quickly when you have a cold?	2.59	0.882	22.5 ^2^	14.8	0.778	0.440
2	find out what you can do so that you don’t get too fat or too thin?	3.21	0.814	12.4 ^2^	41.0	0.663	0.464
3	find out how you can best relax?	3.05	0.864	8.1	33.9	0.746	0.483
4	find out which food is healthy for you?	3.34	0.786	7.2	50.0	0.618	0.424
5	understand when and how you should take your medicine when you are ill?	2.82	0.969	14.8 ^2^	28.1	0.939	0.570
6	understand what your doctor says to you?	2.94	0.849	5.7	26.4	0.721	0.417
7	understand why you sometimes need to see the doctor even though you are not ill?	2.93	0.966	14.8 ^2^	34.3	0.933	0.476
8	understand why you need vaccinations?	2.84	1.08	11.5 ^2^	35.7	1.16	0.590
9	understand what your parents tell you about your health?	3.30	0.791	7.2	47.9	0.625	0.583
10	understand why you need to relax sometimes?	3.38	0.809	5.7	54.3	0.654	0.536
11	judge what helps a lot for you to stay healthy and what does not help much?	3.19	0.811	10.5 ^2^	39.6	0.658	0.654
12	do what your parents tell you to do so that you can get well again?	3.28	0.763	6.2	44.4	0.582	0.432
13	take your medicine in the way you’re told to?	3.08	0.914	9.1	38.9	0.835	0.551
14	stick to what you have learned in road safety lessons?	3.41	0.789	6.2	56.1	0.622	0.450
15	have a healthy diet?	3.53	0.627	5.3	58.6	0.393	0.412

Note. Items translated from Dutch. Abbreviations: SD, standard deviation; ITC, corrected item-total correlations. ^1^ Percentage of participants selecting the maximum possible response option (i.e., “very easy”). ^2^ >10% of participants selected the “do not know” response category.

**Table 3:** Distribution of mean HL scores by quintiles and by HLS-EU-Q47 indices (n = 180).

**Distribution of Mean HL Scores by Quintiles**		
**HL Level**	**n**	**Frequency (%)**
**Lowest HL *(first quintile)***	31	17.2
**Medium HL *(second to fourth quintile)***	110	61.1
**Highest HL *(fifth quintile)***	39	21.7
**Distribution of HL by HLS-EU-Q47 Indices**		
**HL Level**	**n**	**Frequency (%)**
“**Inadequate**” **HL**	17	9.4
“**Problematic**” **HL**	42	23.3
“**Sufficient**” **HL**	82	45.6
“**Excellent**” **HL**	39	21.7

Abbreviations: HL, health literacy.

**Table 4:** Distribution of participants’ overall mean HL scores (n = 180).

Characteristic	n	Mean (SD)	*t*-Value/F-Value	*p*-Value
Sex			1.422	0.157 ^1^
Boys	85	3.19 (0.511)		
Girls	95	3.09 (0.417)		
Age			−2.334	0.021 ^4^
8–9 years	67 ^2^	3.04 (0.453)		
10–11 years	113 ^3^	3.20 (0.463)		
Grade			−2.361	0.019 ^4^
Grade three	80	3.05 (0.465)		
Grade four	100	3.21 (0.455)		
Ethnicity			1.010	0.367
Dutch	131	3.13 (0.436)		
Western	4	3.44 (0.611)		
Non-Western	3	3.06 (0.448)		
SES			0.184	0.832 ^3^
Lowest tertile	23	3.08 (0.574)		
Middle tertile	45	3.13 (0.444)		
Highest tertile	71	3.15 (0.406)		

Abbreviations: SD, standard deviation; SES, socioeconomic status. ^1^ Analyzed by Welch test. ^2^ Eight-year-olds (n = 3) and nine-year-olds (n = 64). ^3^ Ten-year-olds (n = 97) and eleven-year-olds (n = 16). ^4^ Statistically significant difference.

## Data Availability

The data presented in this study are available upon request from the corresponding author. The data are not publicly available as long as data collection in the overall research project is not completed.

## Supplementary Materials

The following are available online at https://www.mdpi.com/article/10.3390/ijerph18105244/s1, Table S1: Inter-item correlations of the HLS-Child-Q15-NL.

## Appendix A. Final Version of the HLS-Child-Q15-NL

**DE VOLGENDE VRAGEN GAAN OVER WAT JE KUNT DOEN OM GEZOND TE BLIJVEN EN OVER WAT JE KUNT DOEN ALS JE ZIEK BENT.**

**Wil jij ons vertellen of de volgende dingen voor jou makkelijk of moeilijk zijn?**

Als je het niet weet of je begrijpt de vraag niet goed, dan kun je “weet niet” antwoorden.

**Zet bij elke zin een kruisje in het hokje dat voor jou klopt.**

Je mag één antwoord per rij geven.

**Hoe makkelijk of moeilijk is het voor jou…**

	**Heel Moeilijk**	**Best Moeilijk**	**Best Makkelijk**	**Heel Makkelijk**	**Weet Niet**
...om erachter te komen hoe je snel beter kunt worden als je verkouden bent?	○	○	○	○	○
...om erachter te komen wat je kunt doen om niet te dik of te dun te worden?	○	○	○	○	○
...om erachter te komen hoe je het beste kunt ontspannen?	○	○	○	○	○
...om erachter te komen welk eten voor jou gezond is?	○	○	○	○	○
...om te begrijpen wanneer en hoe je je medicijnen moet innemen als je ziek bent?	○	○	○	○	○
...om te begrijpen wat de dokter tegen je zegt?	○	○	○	○	○
...om te begrijpen waarom je soms naar de dokter moet, zelfs als je helemaal niet ziek bent?	○	○	○	○	○
...om te begrijpen waarom je moet worden ingeënt (een prik krijgt)?	○	○	○	○	○
...om te begrijpen wat je ouders je vertellen over je gezondheid?	○	○	○	○	○
...om te begrijpen waarom je soms ook moet uitrusten?	○	○	○	○	○
...om te kiezen wat voor jou wel en niet helpt om gezond te blijven?	○	○	○	○	○
...om te doen wat je ouders tegen je zeggen om weer beter te worden?	○	○	○	○	○
...om je medicijnen in te nemen zoals het je is verteld?	○	○	○	○	○
...om je te houden aan de verkeersregels die je hebt geleerd?	○	○	○	○	○
	**Heel moeilijk**	**Best moeilijk**	**Best makkelijk**	**Heel makkelijk**	**Weet niet**
...om gezond te eten?	○	○	○	○	○
