# Supplementary material for: Measuring and Exploring Children’s Health Literacy in The Netherlands: Translation and Adaptation of the HLS-Child-Q15
Source: Int J Environ Res Public Health. 2021 May 14;18(10):5244. doi: 10.3390/ijerph18105244 (PMC8156463; doi:10.3390/ijerph18105244)
Supplement: Supplementary file 1 [file ijerph-18-05244-s001.zip › ijerph-1196278-supplementary.pdf]

Table S1. Inter-item correlations of the HLS-Child-Q15-NL.

Inter-Item Correlation Matrix															
	**Item 1**	**Item 2**	**Item 3**	**Item 4**	**Item 5**	**Item 6**	**Item 7**	**Item 8**	**Item 9**	**Item 10**	**Item 11**	**Item 12**	**Item 13**	**Item 14**	**Item 15**
**Item 1**	1.000	0.461	0.299	0.115	0.336	0.166	0.275	0.413	0.191	0.223	0.341	0.151	0.358	0.130	0.104
**Item 2**	0.461	1.000	0.311	0.338	0.310	0.091	0.208	0.292	0.171	0.188	0.436	0.198	0.238	0.226	0.392
**Item 3**	0.299	0.311	1.000	0.258	0.237	0.093	0.252	0.328	0.400	0.368	0.309	0.209	0.391	0.212	0.298
**Item 4**	0.115	0.338	0.258	1.000	0.295	0.117	0.250	0.226	0.419	0.120	0.558	0.009	0.132	0.290	0.411
**Item 5**	0.336	0.310	0.237	0.295	1.000	0.284	0.368	0.456	0.330	0.373	0.333	0.226	0.472	0.275	0.290
**Item 6**	0.166	0.091	0.093	0.117	0.284	1.000	0.301	0.264	0.340	0.431	0.345	0.331	0.266	0.232	0.182

	Item 1	Item 2	Item 3	Item 4	Item 5	Item 6	Item 7	Item 8	Item 9	Item 10	Item 11	Item 12	Item 13	Item 14	Item 15
**Item 7**	0.275	0.208	0.252	0.250	0.368	0.301	1.000	0.441	0.262	0.336	0.327	0.208	0.302	0.266	0.025
**Item 8**	0.413	0.292	0.328	0.226	0.456	0.264	0.441	1.000	0.370	0.403	0.356	0.324	0.358	0.310	0.179
**Item 9**	0.191	0.171	0.400	0.419	0.330	0.340	0.262	0.370	1.000	0.455	0.523	0.444	0.369	0.201	0.278
**Item 10**	0.223	0.188	0.368	0.120	0.373	0.431	0.336	0.403	0.455	1.000	0.344	0.348	0.262	0.365	0.110
**Item 11**	0.341	0.436	0.309	0.558	0.333	0.345	0.327	0.356	0.523	0.344	1.000	0.282	0.263	0.391	0.508
**Item 12**	0.151	0.198	0.209	0.009	0.226	0.331	0.208	0.324	0.444	0.348	0.282	1.000	0.388	0.207	0.249

	Item 1	Item 2	Item 3	Item 4	Item 5	Item 6	Item 7	Item 8	Item 9	Item 10	Item 11	Item 12	Item 13	Item 14	Item 15
**Item 13**	0.358	0.238	0.391	0.132	0.472	0.266	0.302	0.358	0.369	0.262	0.263	0.388	1.000	0.419	0.277
**Item 14**	0.130	0.226	0.212	0.290	0.275	0.232	0.266	0.310	0.201	0.365	0.391	0.207	0.419	1.000	0.185
**Item 15**	0.104	0.392	0.298	0.411	0.290	0.182	0.025	0.179	0.278	0.110	0.508	0.249	0.277	0.185	1.000
